# The Impact of Artificial Intelligence and Deep Learning in Eye Diseases: A Review

**DOI:** 10.3389/fmed.2021.710329

**Published:** 2021-08-30

**Authors:** Raffaele Nuzzi, Giacomo Boscia, Paola Marolo, Federico Ricardi

**Affiliations:** Ophthalmology Unit, A.O.U. City of Health and Science of Turin, Department of Surgical Sciences, University of Turin, Turin, Italy

**Keywords:** artificial intelligence, machine learning, deep learning, neural network, teleophthalmology, ophthalmology

## Abstract

Artificial intelligence (AI) is a subset of computer science dealing with the development and training of algorithms that try to replicate human intelligence. We report a clinical overview of the basic principles of AI that are fundamental to appreciating its application to ophthalmology practice. Here, we review the most common eye diseases, focusing on some of the potential challenges and limitations emerging with the development and application of this new technology into ophthalmology.

## Introduction

In the near future, the number of patients suffering from eye diseases is expected to increase dramatically due to aging of the population. In such a scenario, early recognition and correct management of eye diseases are the main objectives to preserve vision and enhance quality of life. Deep integration of artificial intelligence (AI) in ophthalmology may be helpful at this aim, having the potential to speed up the diagnostic process and to reduce the human resources required. AI is a subset of computer science that deals with using computers to develop algorithms that try to simulate human intelligence.

The concept of AI was first introduced in 1956 (1). Since then, the field has made remarkable progress to the point that it has been defined as “the fourth industrial revolution in mankind's history” (2).

**Figure 1:**
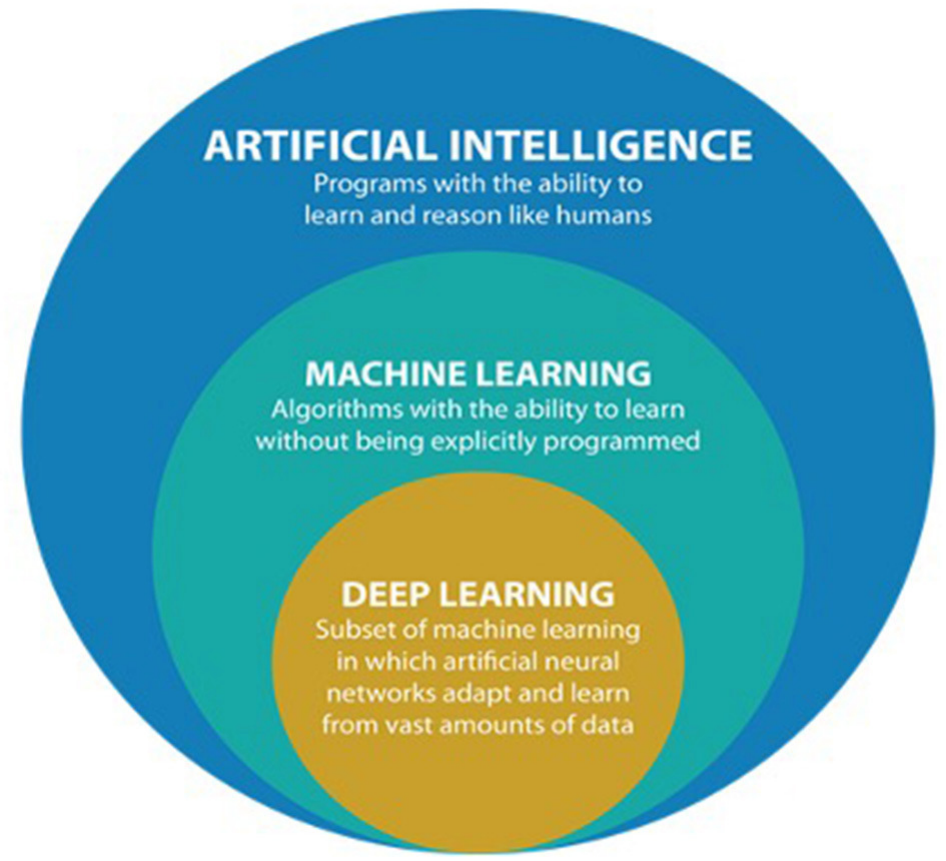
Comprehensive overview of artificial intelligence (AI) and its subfields (from https://datacatchup.com/artificial-intelligence-machine-learning-and-deep-learning/).

The terms artificial intelligence, machine learning, and deep learning (DL) have been used at times as synonyms; however, it is important to distinguish the three (Figure 1).

Artificial intelligence is the most general term, referring to the “development of computer systems able to perform tasks by mimicking human intelligence, such as visual perception, decision making, and voice recognition” (3). Machine learning, which occurred in the 1980s, refers to a subfield of AI that allows computers to improve at performing tasks with experience or to “learn on their own without being explicitly programmed” (4).

**Figure 2:**
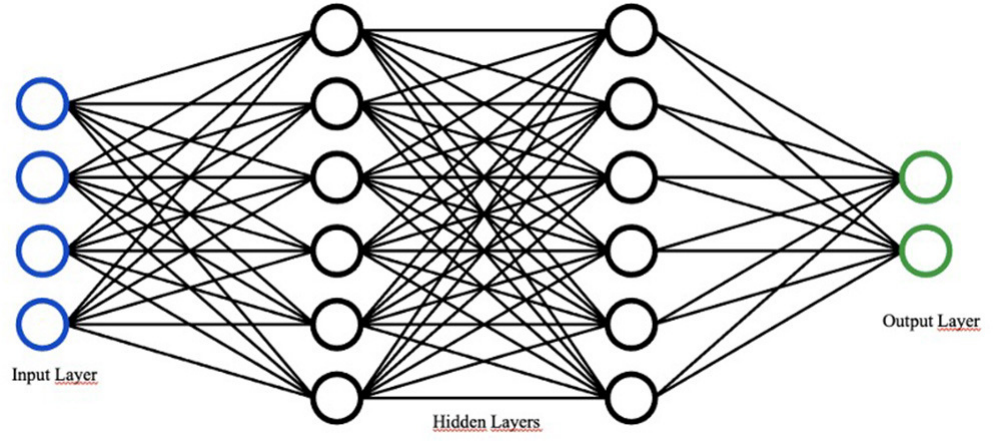
Basic design of a neural network. Adapted from “Network.svg” by Victor C. Zhou (https://victorzhou.com/series/neural-networks-from-scratch/).

Finally, deep learning refers to a “subfield of machine learning composed of algorithms that use a cascade of multilayered artificial neural networks for feature extraction and transformation” (5, 6). The term “deep” refers to the many deep hidden layers in its neural network: the benefit of having more layers of analysis is the ability to analyze more complicated inputs, including entire images. In other words, DL uses representation learning methods with multiple levels of abstraction to elaborate and process input data and generate outputs without the need for manual feature engineering, automatically recognizing the intricate structures embedded in high-dimensional data (7) (Figure 2).

The entire field of healthcare has been revolutionized by the application of AI to the current clinical workflow, including in the analysis of breast histopathology specimens (8), skin cancer classification (9), cardiovascular risk prediction (10), and lung cancer detection (11). This expanding research inspired numerous studies of AI application also to ophthalmology, leading to the development of advanced AI algorithms together with multiple accessible datasets such as EyePACS (12), Messidor (12), and Kaggle's dataset (13).

Deep learning has been largely reported to be capable of achieving automated screening and diagnosis of common vision-threatening diseases, such as diabetic retinopathy (DR), glaucoma, age-related macular degeneration (AMD), and retinopathy of prematurity (ROP).

Further integration of DL into ophthalmology clinical practice is expected to innovate and improve the current disease and management process, including an earlier detection and hopefully better disease outcomes.

## Materials and Methods

The outcome of this review was to provide a descriptive analysis of the current and most clinically relevant applications of AI in the various fields of ophthalmology. A literature search was conducted by two independent investigators (FR and GB), from the earliest available year of indexing until February 28, 2021. Two databases were used during the literature search: MEDLINE and Scopus. The following terms were connected using the Boolean operators “and,” “or,” “and/or”: “Artificial Intelligence,” “Ophthalmology,” “Diabetic Retinopathy,” “Age Related Macular Degeneration,” “Retinal Detachment,” “Retinal Vein Occlusion,” “Cataract,” “Keratoconus,” “Glaucoma,” “Pediatric Ophthalmology,” “Retinopathy of Prematurity,” “Teleophthalmology,” “Eyelid Tumors,” “Exophthalmos,” and “Strabismus.” The terms were searched as “Mesh terms” and as “All fields” terms. No limitations were placed on the keyword searches. Full articles or abstracts that were written in English were included. Only articles published in peer-reviewed journals were selected in this review. The investigators screened the search results and selected the most recent and noteworthy publications with such an impact on clinical practice. In particular, the inclusion criteria were: a clear methodology of algorithm development and training, high number of images and/or data used for training the DL algorithm, and high rates of disease prediction/detection in terms of sensibility, specificity, and area under the receiver operating characteristic curve (AUC).

Data extracted from each selected paper included: the first author of the study, year, time frame, study design, location, follow-up time, number of eyes enrolled, demographic features (mean age, gender, and ethnicity), ophthalmological pathology under investigation (DR, AMD, retinal detachment, retinal vein occlusion, cataract, keratoconus, glaucoma, ROP, pediatric cataract, strabismus, myopia, and teleophthalmology), and AI characteristics (imaging type, disease definition, sensitivity, specificity, and accuracy).

## Results

### Study Selection

The review included a total of 69 studies, of which one study was about exophthalmos (13), three about strabismus (14–16), two studies about eyelid tumors (17, 18), three about keratoconus (19–21), seven about cataracts (22–28), three about pediatric cataracts (29–31), one about myopia (32), nine about glaucoma (33–41), nine studies were about DR (11, 34, 39, 42–47), nine about AMD (34, 48–55), two about retinal detachment (56, 57), one about retinal vein occlusion (58), 12 about ROP (59–70), and seven were about teleophthalmology (71–77).

### Exophthalmos

One of the most common causes of exophthalmos is the thyroid-associated ophthalmopathy (TAO). Salvi et al. (14) developed a model to evaluate the disease classification and prediction of progression. They considered a group of 246 patients with absent, minimal, or inactive TAO and 152 patients with progressive and/or active TAO. The research collected variations of 13 clinical eye signs. The neural network they used obtained a concordance with clinical assessment of 67%.

### Strabismus

Lu et al. (15) used a convolutional neural network (CNN) together with facial photos to detect abnormal position of the eye. This could be beneficial in telemedical evaluation and screening. On the contrary, for in-office evaluation, the CNN could be applied to eye-tracking data (16) or to retinal birefringence scanning (17).

### Eyelid Tumors

Wang et al. (18), using a DL (VGG16) that contained the parameters learnt from ImageNet2014, developed a protocol to classify eyelid tumors by distinguishing digital pathological slides that have either malignant melanoma or non-malignant melanoma at the small patch level. They used 79 formalin-fixed paraffin-embedded pathological slides from 73 patients, divided into 55 non-malignant melanoma slides from 55 patients and 24 malignant slides from 18 patients cut into patches. The validation consisted of 142,104 patches from 79 slides, with 61,031 non-malignant patches from 55 slides and 81,073 malignant patches from 24 slides. The AUC for the algorithm was 0.989.

Tan et al. (19) developed a model to predict the complexity of reconstructive surgery after periocular basal cell carcinoma excision. The three predictive variables were preoperative assessment of complexity, surgical delays, and tumor size. They obtained an AUC of 0.853.

### Keratoconus

The most important diagnostic imaging techniques for keratoconus include corneal topography with a Placido disc-based imaging system (Orbscan, Bausch & Lomb, Bridgewater, NJ, USA), anterior segment optical coherence tomography (AS-OCT), and three-dimensional (3D) tomographic imaging, such as Scheimpflug (Pentacam, Oculus, Lynnwood, WA, USA). On this basis, Yousefi et al. developed an unsupervised machine learning algorithm for the grading of keratoconus using 3,156 AS-OCT images of keratoconus from grade 0 to grade 4. It showed a sensibility of 97.7% and a specificity of 94.1% (20). In 2019, Kamiya et al. reported higher sensibility (99.1%) and specificity (98.4%) for keratoconus grading with a CNN that used 304 AS-OCT images of keratoconus from grade 0 to grade 4 (21). Finally, Lavric and Valentin developed a CNN trained using 1,350 healthy eye and 1,350 keratoconus eye topographies, with a validation set of 150 eyes, that showed an accuracy of 99.3% (78).

### Cataract

The AI technology has been applied to various aspects of cataract, both on clinical and surgical management, from diagnosing cataracts to optimizing the biometry for intraocular lens (IOL) power calculation.

The clinical classification of cataracts includes nuclear sclerotic, cortical, and posterior subcapsular. These are usually diagnosed by slit lamp microscopy and/or photography. Cataract is graded on clinical scales such as the Lens Opacities Classification System III (22). One of the first AI systems for evaluating nuclear cataracts was described by Li et al. in 2009. Their system had a success rate of 95% (23). Xu et al., in 2013, evaluated an automatic grading method of nuclear cataracts from slit lamp lens images using group sparsity regression, obtaining a mean absolute error of 0.336 (24). In 2015, Gao et al. (25) used 5,378 slit lamp photographs to develop an algorithm to grade nuclear cataracts, obtaining an accuracy of 70.7%. In a recent large-scale study, Wu et al. (26), in China, used DL *via* residual neural network (ResNet) to establish a three-step sequential AI algorithm for the diagnosis of cataracts. This algorithm was trained with 37,638 slit lamp photographs in order to differentiate cataract and IOL from normal lens (AUC > 0.99) and to detect referable cataracts (AUC > 0.91).

With the increasing use of retinal imaging, other researchers have also explored the use of color fundus photographs for the development of an automated cataract evaluation system, potentially leveraging on retinal imaging as an opportunistic screening tool for cataracts as well. Dong et al. (27) developed an AI algorithm with a combination of machine learning and DL using 5,495 fundus images. The goal was to describe a classification of the “visibility” of fundus images to report four classes of cataract severity (normal, mild, moderate, and severe). The accuracy was 94.07%. Zhang et al., in 2017, proposed a system to classify cataracts, obtaining an accuracy of 93.52% (28). Li et al., in 2018, published an article reporting accuracies of 97.2 and 87.7%, respectively, for detecting and grading tasks (29). Ran et al. (30) proposed a six-level cataract grading based on the feature datasets generated by a deep convolutional neural network (DCNN). Xu et al. (31) have developed CNN-based algorithms, AlexNet and VisualDN, with the purpose of diagnosing and grading cataracts, gaining an accuracy of 86.2% using 8,030 fundus images. Pratap and Kokil, in 2019, trained a CNN for automatic cataract classification, obtaining an accuracy of 92.91% (79). Zhang et al. (32) showed a higher accuracy of 93% in the detection and grading of cataracts using 1,352 fundus images.

Currently, the choice of an IOL power calculation formula remains unstandardized and at the discretion of the surgeon. Ocular parameters such as axial length and keratometry are important factors when determining the applicability of each formula. In 2015, with the introduction of a concept of an IOL, “Ladas super formula” (80), the method of IOL calculation changed radically. Previous generations of IOL formulas were developed as 2D algorithms. This new methodology was derived by extracting features from respective “ideal parts” of old formulas (Hoffer Q, Holladay-1, Holladay-1 with Koch adjustment, Haigis, and SRK/T, with the exclusion of the Barrett Universal II and Barrett Toric formulas) and plotting into a 3D surface. The super formula may serve as a solution to calculating eyes with typical and atypical parameters such as axial length, corneal power, and anterior chamber depth. The concept of three-dimensionality is to develop a way to compare one or more formulas, allowing for the evaluation of areas of clinical agreements and disagreements between multiple formulas (81). Recently, Kane et al. have demonstrated a Hill-RBF (radial basis function) method using a large dataset with an adaptive learning to calculate the refractive output. For a given eye, it relies on adequate numbers of eyes of similar dimensions to provide an accurate prediction (82).

### Pediatric Cataract

Pediatric cataract is a more variable disease than are cataracts developing in adults. Moreover, slit lamp examination and cataract visualization could be challenging because these are based on child compliance. CC-Cruiser (33–35) is a cloud-based system that can automatically identify cataracts from slit lamp images, grade them, and suggest treatment. Although this approach could lead to a higher patient satisfaction due to its rapid evaluation, it is characterized by a significantly lower performance in diagnosing cataracts and in recommending treatment than by experts (34).

### High Myopia

Children at risk of high myopia could benefit from assuming low-dose atropine to stop or slow down myopic progression (36); however, determining for which children this therapy should be prescribed can be challenging (37). For this reason, Lin et al. (37) tried to predict high-grade myopia progression in children using a clinical measure, showing good predictive performance for up to 8 years in the future. This approach could represent a better guide to prophylactic treatment.

### Glaucoma

The main difficulty in detecting and treating glaucoma consists in its being asymptomatic at the early stages (38). In this scenario, AI can be helpful in detecting the glaucomatous disc, interpreting the visual field tests, and forecasting clinical outcomes (39).

Given the dissimilarity in optic disc anatomy, identifying the glaucomatous optic nerve head (ONH) can be difficult at the early stages of the disease. Moreover, it was shown that, even among experts, agreement on the detection of ONH damage is modest (83). The difference in identifying the glaucomatous disc on fundus photographs is magnified by variations in the image capturing device, mydriasis state, focus, and exposure. Given that, AI can implement different sources of data and help in defining ONH damage. Some investigators have trained DL algorithms to detect a cup/disc ratio (CDR) at or above a certain threshold (either a CDR of 0.7 or 0.8) with AUC ≥ 0.942 (40). Ting et al., in 2017, developed an algorithm through a dataset of retinal images for the detection of DR, glaucoma, and AMD on a multiethnic population. In particular, for glaucoma detection, their algorithm presented an AUC of 0.942, sensibility of 96.4%, and specificity of 87.2% (41). Using a different approach, the investigators defined the glaucoma status by linking other data with the optic disc photograph, obtaining remarkably good results (AUC ≥ 0.872) (42, 84–86). Moreover, Asaoka et al. applied DL to OCT images, obtaining an even higher AUC (0.937) than did other machine learning methods (43). Finally, Medeiros et al. used an innovative approach, training a DL algorithm from OCT scans to predict retinal nerve fiber layer (RNFL) thickness on fundus photos, with a high correlation of prediction of 0.83 and an AUC of 0.944 (85) (Table 1).

**Table 1:** Summary of studies on AI and glaucoma.

**Reference**	**Imaging**	**No. of images**	**Disease definition**	**Sensitivity**	**Specificity**	**AUC**
Ting et al. (41)	Fundus images	125,189	CDR, 0.8+	0.964	0.872	0.942
Li et al. (40)	Fundus images	48,116	CDR, 0.7+	0.956	0.920	0.986
Shibata et al. (84)	Fundus images	3,620	Glaucoma	ns	ns	0.965
Masumoto et al. (86)	Fundus images	1,399	Glaucoma	0.813	0.802	0.872
Medeiros et al. (85)	Fundus images and OCT scans	32,820	Glaucomatous visual field loss	ns	ns	0.944
Thompson et al. (42)	Fundus images and OCT scans	9,282	Glaucomatous visual field loss	ns	ns	0.933
Asaoka et al. (43)	OCT	2,132	Early glaucoma	0.825	0.939	0.937

*AI, artificial intelligence; OCT, optical coherence tomography; CDR, cup/disc ratio; AUC, area under the receiver operating characteristic curve; ns, not specified*.

Visual fields, unlike fundus photographs and OCT scans, represent the functional assay of the visual pathway. Despite being a fundamental exam in clinical evaluation, current algorithms applied to visual fields do not differentiate subtle loss in a regional manner and glaucomatous from the non-glaucomatous defects and artifacts (39). Moreover, the current computerized packages do not decompose visual field data into patterns of loss. Visual field loss patterns are due to the compromised RNFs projecting to specific areas of the optic disc. Recently, Elze et al. (44) have developed an unsupervised algorithm “employing a corner learning strategy called archetypal analysis to quantitatively classify the regional patterns of loss without the potential bias of clinical experience”.

Archetypal analysis provides a regional stratification of the visual fields together with the coefficients weighting each pattern loss. Furthermore, implementation of AI algorithms to visual field testing could also assist clinicians in tracking the visual field progression with more accuracy (45). Finally, in more recent years, AI has been used to forecast glaucoma progression using Kalman filtering. This technique could lead to the generation of a personalized disease prediction based on different sources of data, which can help clinicians in the decision-making process (46, 47).

### Diabetic Retinopathy

Several studies have implemented DL algorithms for the diagnosis of microaneurysms, hemorrhages, hard exudates, and cotton wool spots among patients with DR. DL algorithms for the detection of DR have recently been reported to have a higher sensitivity than does manual detection by ophthalmologists (48). However, more studies are needed to confirm this thesis (12).

The accuracy of AI depends on access to good training datasets. Gulshan et al. (12) evaluated the accuracy of a two-dataset system in the detection of DR from fundus photographs: the EyePACS-1 dataset, composed of 9,963 images from 4,997 patients, and the MESSIDOR-2 dataset, consisting of 1,748 images from 874 people. One of the earliest studies on the automated detection of DR from color fundus photographs was by Abramoff et al. in 2008 (49). It was a retrospective analysis done with non-mydriatic images that was able to detect referable DR with 84% sensitivity and 64% specificity. In 2013 (50), in a research showing the results of the Iowa Detection Program, a higher sensitivity of 96.8% and a lower specificity of 59.4% were found. Another study published in 2015, using EyeArt AI software trained with the MESSIDOR-2 dataset, demonstrated a sensitivity of 93.3% and a specificity of 72.2% in diagnosing DR (51). Later, in 2016, Gulshan et al. developed an algorithm trained with 128,175 macula-centered retinal images obtained from EyePACS and MESSIDOR-2 and reported sensitivity values of 97.5 and 96.1% and specificities of 93.4 and 93.3%, respectively (12).

Gargeya and Leng (52) focused on the identification of mild non-proliferative DR. They showed a sensitivity of 94% and a specificity of 98% for referable DR with EyePACS.

As reported above, Ting et al. developed an algorithm through a dataset of retinal images for the detection of DR, glaucoma, and AMD on a multiethnic population. This DL system evaluated 76,370 retinal images with a sensitivity of 90.5% and a specificity of 91.6% for DR (41). Ardiyanto et al. (53) developed an algorithm for DR grading trained with 315 fundus images, with an accuracy of 95.71%, a sensitivity of 76.92%, and a specificity of 100%. Takahashi et al. (54) proposed a novel AI disease staging system with the ability to grade DR involving retinal areas not typically visualized on fundoscopy. They obtained an algorithm able to grade DR with 9,939 fundus images with an accuracy of 64–82%. Finally, Rajalakshimi et al. (55) showed the possibility of a smartphone-based fundus image diagnosis of DR with a sensitivity of 95.8% and a specificity of 66.8% (Table 2).

**Table 2:** Summary of studies about AI and diabetic retinopathy.

**Reference**	**Imaging**	**No. of images**	**Disease definition**	**Sensitivity**	**Specificity**	**AUC**	**Accuracy**
Abramoff et al. (50)	Fundus photos	1,748	Detection of DR	0.968	0.594	0.937	ns
Solanki et al. (51)	Fundus photos	755	Detection of DR	0.933	0.722	0.965	ns
Gulshan et al. (12)	Fundus photos	136,886	Evaluation of DR	EYEPACS-1: 0.975 Messidor-2: 0.961	EYEPACS-1: 0.934 Messidor-2: 0.939	0.991	ns
Gargeya et al. (52)	Fundus photos	76,885	Evaluation of DR	0.94	0.98	0.94–0.97	ns
Rajalakshimi et al. (55)	Smartphone-based fundus images	2,048	Detection of DR	0.958	0.668	ns	ns
Ardiyanto et al. (53)	Fundus photos	315	Grade of DR	0.7692	1.0	ns	95.71%
Takahashi et al. (54)	Fundus photos	9,936	Grade of DR	ns	ns	ns	64%−82%
Ting et al. (41)	Fundus photos	76,370	Detection of DR	0.905	0.916	0.936	ns

*AI, artificial intelligence; AUC, area under the receiver operating characteristic curve; DR, diabetic retinopathy; ns, not specified*.

### Age-Related Macular Degeneration

Several studies have used fundus photographs in the diagnosis of AMD. Burlina et al. (56) focused on the automated grading of AMD. From color fundus images, they evaluated the classification between absence/early AMD and intermediate/advanced AMD, with an accuracy of 0.94–0.96. Treder et al. (57) focused on a DL-based detection and classification of geographic atrophy using a DCNN classifier through autofluorescence fundus photos. They obtained an accuracy of 91–96%. Another study (58) used a deep, unspecified CNN to classify between normal and wet AMD images, with a sensitivity of 100%, a specificity of 97.31%, and an accuracy of 99.76%. Two hundred fifty-three fundus photos for training and 111 for validation were used. Keel et al. (59) developed a DL algorithm for the detection of neovascular AMD using color fundus photographs, with a sensitivity of 96.7%, a specificity of 96.4%, and an accuracy of 99.5%. Ting et al. (41) showed an AUC for their algorithm of 0.931, a sensitivity of 93.2%, and a specificity of 88.4% when compared to manual efforts by ophthalmologists.

Concerning OCT, Bogunovic et al. developed a data-driven model to predict the progression risk in intermediate AMD (60). Another research in 2017 developed an algorithm to predict anti-vascular endothelial growth factor (VEGF) treatment requirements in neovascular AMD. They used quantitative OCT scan features to classify the need for injections over 20 months into high (more than 15), medium (between 6 and 15), and low (<6) groups and used the OCT images of 317 patients as a dataset for training and validation (61). An accuracy of 70%−80% was achieved for treatment requirement evaluation. Treder et al., in 2017, established a model able to detect automatically exudative AMD from spectral domain OCT (SD-OCT) (57). Prahs et al. (62) developed an OCT-based DL algorithm for the evaluation of treatment indication with anti-VEGF. This research included a deep, unsupervised CNN to compare the system prediction injection requirement to actual injection administration within 21 days. This kind of AI used more than 150,000 OCT line scans for training and 5,358 for validation, with an AUC of 96.8%, a sensitivity of 90.1%, and a specificity of 96.2%. Sengupta et al. (63) used OCT images with the aim of differentiating between AMD and diabetic macular edema, obtaining a sensitivity of 97.8% and a specificity of 97.4% (Table 3).

**Table 3:** Summary of studies about AI and age-related macular degeneration.

**Reference**	**Imaging**	**No. of images**	**Disease definition**	**Sensitivity**	**Specificity**	**AUC**	**Accuracy**
Burlina et al. (56)	Color fundus photos	>130,000	Grading AMD	0.884	0.941	0.94–0.96	ns
Treder et al. (57)	Autofluorescence fundus photos	600	Classification of geographic atrophy	1	0.92	ns	91%−96%
Matsuba et al. (58)	Fundus photos	253	AMD normal *vs*. wet	1	0.9731	0.9976	ns
Keel et al. (59)	Fundus photos	27,397	Neovascular AMD	0.967	0.964	0.995	ns
Bogunovic et al. (60)	OCT images	317	Treatment with anti-VEGF	ns	ns	0.70–0.80	ns
Prahs et al. (62)	OCT images	153,912	Treatment with anti-VEGF	0.901	0.962	0.968	ns
Ting et al. (41)	Fundus photos	35,948	Detection AMD	0.932	0.884	0.931	ns
Sengupta et al. (63)	OCT images	Normal: 51,140 Drusen: 8,617 CNV: 37,206 DME: 11,349	Differentiate AMD and DME	0.978	0.974	ns	ns

*AI, artificial intelligence; OCT, optical coherence tomography; CNV, choroidal neovascularization; DME, diabetic macular edema; AMD, age-related macular degeneration; VEGF, vascular endothelial growth factor; ns, not specified*.

### Retinal Detachment

Ohsugi et al. (64) used fundus ophthalmoscopy to detect retinal detachment, with 329 fundus photos for training and 82 for validation, obtaining a sensitivity of 97.6.7%, a specificity of 96.5%, and an accuracy of 99.6%. Li et al. (65) developed a deep, unsupervised CNN to detect retinal detachment and macula-on status through 7,323 fundus photos for training and 1,556 for validation. The sensitivity values were 96.1 and 93.8%, the specificities were 99.6% and 90.9%, and the AUCs were 0.989 and 0.975, respectively.

### Retinal Vein Occlusion

One of the most important researches on retina vein occlusion was that by Zhao et al. (66), who used a CNN together with patch-based and image-based vote methods to identify the fundus image of branch retinal vein occlusion automatically. They achieved an accuracy of 97%.

### Retinopathy of Prematurity

The most significant progresses in the pediatric application of AI deal with ROP. Automation derived from AI application could not only improve screening and objective assessment but also cause less stress and pain for infants undergoing examination compared with indirect ophthalmoscopy (67). Different studies have focused on the determination of the vessel tortuosity and width *via* fundus images, creating tools like Vessel Finder (68), Vessel Map (69), ROP tool (70), Retinal Image Multiscale Analysis (RISA) (71, 87), and Computer-Aided Image Analysis of the Retina (CAIAR) (72, 73). Vessel measurements were used as a feature for various predictive models of diseases. Finally, recent approaches to ROP are mostly based on CNNs, which take fundus images as inputs and do not require manual intervention. Systems like that of Worral and Wilson (74), the i-ROP-DL (75, 76), and DeepROP (77) have demonstrated agreement with expert opinions and better disease detection than that by some experts (76, 77).

### Teleophthalmology and Screening

**Figure 3:**
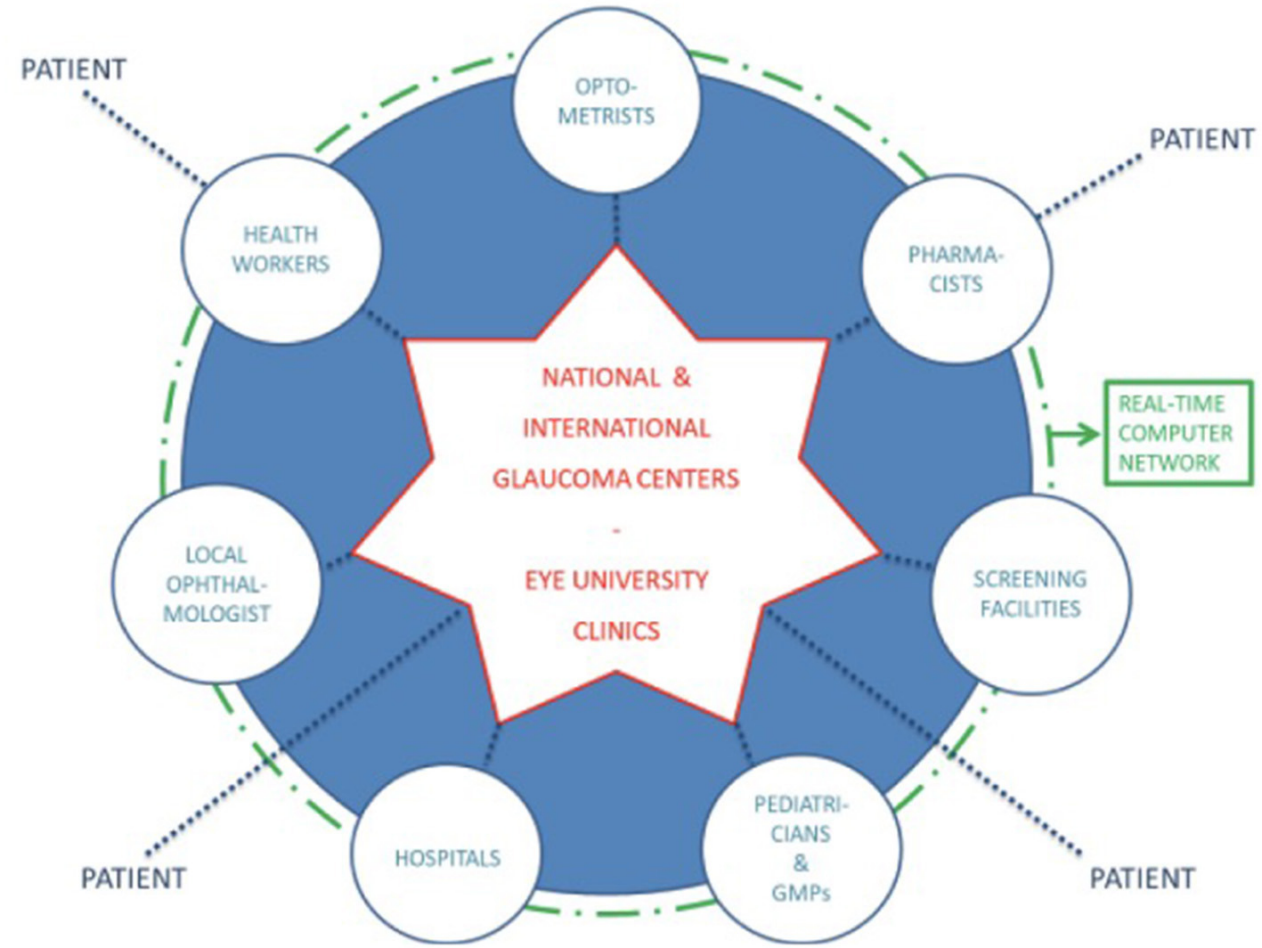
The hub and spoke model of glaucoma (89).

Telemedicine is defined as “the use of medical information exchanged from one site to another *via* electronic communications to improve a patient's health status” (88). Telemedicine can facilitate a larger distribution of healthcare to distant areas where there is a lack of health workers, can reduce the waiting times, and improve acute management of patients, even in remote regions. A possible example of the application of telemedicine to glaucoma management is represented by the “hub and spoke pre-hospital model of glaucoma: the hubs correspond to optometrists, pharmacists, pediatricians, general medical practitioners (GMPs), the local ophthalmologist, health workers, screening facilities, and hospitals, while the spoke is represented by the national and international glaucoma centers and the Eye University Clinics” (89) (Figure 3). This model of healthcare improves and expands clinical services to remote regions (the so-called spoke) with consultations between patients and specialists based in referral centers (the so-called hub) (90).

However, given the global burden of eye diseases and the progressive shortage of healthcare workers, telemedicine solutions that decentralize consultations could not be sufficient.

In such a scenario, the AI technology should be combined with telemedicine, enabling clinicians to receive automatically acquired and screened health parameters from the patients and to visit them remotely. Especially during the coronavirus disease 2019 (COVID-19) pandemic era, the implementation of teleophthalmology could reduce the infection risk in the healthcare setting, enabling remote triaging before arriving to the hospital in order to avoid unnecessary visits and exposure risks, as already done by multiple centers across the world (91–94).

Furthermore, the DL algorithm applied to retinal fundus images could be useful as a broad-based visual impairment screening tool, as reported by Tham et al. (95). This approach could lead to a more rapid referral of patients with visual impairments to tertiary eye centers.

Another step forward in improving traditional clinic visits might be further accelerated by the application of AI to home devices used for monitoring parameters such as visual acuity, visual fields, and intraocular pressure (96–99). Although further studies need to be conducted before the mass adoption of these devices, they provide assurance of the possibility to utilize teleophthalmology from home in the future.

## Discussion

In this review, we described the main applications of AI to ophthalmology, underlining many aspects of evolution and future improvements related to this technology.

Several other reviews on AI in ophthalmology have been published (100); however, they are more focused on specific diseases such as DR or AMD (101–103). Although these articles are exhaustive and are in-depth reviews, the aim of our work was to create a more comprehensive clinical overview of the current applications of AI in ophthalmology, giving the clinicians a practical summary of current evidence for AI.

DL algorithms reached high thresholds of accuracy, sensitivity, and specificity for some of the most common vision-threatening diseases, with the highest level of evidence regarding DR, AMD, and glaucoma. Furthermore, some studies on AI have focused on pediatric ophthalmology, with the aim of helping clinicians in overcoming common practical limitations due to pediatric patients' compliance.

However, in such a scenario of optimistic acceptance of this new technology, it should be highlighted that DL has also given rise to challenges and some controversy.

Firstly, DL algorithms are poorly explainable in ophthalmology terms. This is the so-called black box phenomenon and could eventually lead to a reduced acceptance of this technology by clinicians (104, 105). A black box suggests lack of understanding of the decision-making process of the algorithm that gives a certain output. Several techniques are used to bound this phenomenon, such as the “occlusion test, in which a blank area is systemically moved across the entire image and the largest drop in predictive probability represents the specific area of highest importance for the algorithm” (106), or “saliency maps (heat maps) generation techniques such as activation mapping, which, again, highlights areas of importance for classification decisions within an image” (107). Despite the progress made, in some cases (108), the visualization method highlighted non-traditional areas of diagnostic interest, and it is uncertain how to consider the features identified by saliency analysis of those regions (109).

External validation of algorithms represents the second challenge. Although many DL algorithms have been developed based on publicly available datasets, there are some concerns about how well these will perform in a “real-world” clinical practice setting (109, 110). When adopted in clinical practice, these algorithms may have a diminished performance due to variabilities in several aspects, such as in the imaging quality, lighting conditions, and the different dilation protocols.

Another area of controversy is the presence of *bias* in the datasets for training algorithms. Biases in the training data used for developing AI algorithms not only may weaken the external applicability but may also amplify preexisting biases (109). Some solutions to recognize potential biases and limit unwanted outcomes could be to rebalance the training dataset if a certain subgroup is underrepresented or to select a training dataset with diverse patient populations. An example of an adequate training dataset on different populations is that used by Ting et al. (41), in which their algorithm for detecting diabetic retinopathy was validated on different ethnic groups.

Lastly, it is relevant noticing the legal implications of using DL algorithms in clinical practice. In fact, if a machine thinks similarly to a human ophthalmologist who can make errors, “who is responsible to bear the legal consequences of an undesirable outcome due to an erroneous prediction made by an artificial intelligence algorithm? These are such complex medical legal issues that have yet to be settled” (109, 111).

## Conclusions

In conclusion, we discussed the main fields for the application of AI into ophthalmology practice. The use of AI algorithms should be seen as a tool to assist clinicians, not to replace them. AI could speed up some processes, reduce the workload for clinicians, and minimize diagnostic errors due to inappropriate data integration. AI is able to extract features from complex and different imaging modalities, enabling the discovery of new biomarkers to expand our current knowledge of diseases. This could lead to introducing into clinical practice new automatically detected diagnostic parameters or to developing new treatments for eye diseases. Challenges related to the implementation of these technologies remain, including algorithm validation, patient acceptance, and the education and training of health providers. However, physicians should continue to adapt to the fast-changing models of care delivery, collaborating more with teams of engineers, data scientists, and technology experts in order to achieve high-quality standards for research and interdisciplinary clinical practice.

## Author Contributions

RN, GB, PM, and FR have contributed to manuscript drafting, literature review, and final approval of the review. All authors contributed to the article and approved the submitted version.

## Conflict of Interest

The authors declare that the research was conducted in the absence of any commercial or financial relationships that could be construed as a potential conflict of interest.

## Publisher's Note

All claims expressed in this article are solely those of the authors and do not necessarily represent those of their affiliated organizations, or those of the publisher, the editors and the reviewers. Any product that may be evaluated in this article, or claim that may be made by its manufacturer, is not guaranteed or endorsed by the publisher.
